# Staying in the Room

**DOI:** 10.5811/westjem.2015.9.28563

**Published:** 2015-12-01

**Authors:** Jesse Z. Kellar

**Affiliations:** Lakeland Health Emergency Medicine Residency, Department of Emergency Medicine, St Joseph, Michigan

The call from the nursing home relayed that an 85-year-old male was coming in by ambulance complaining of increased shortness of breath. The nursing home told us that he had an unknown code status. Once the patient arrived, it was clear that he was in the process of dying. While I was preparing for aggressive resuscitative efforts, my attending physician was shuffling through some paperwork that came with the patient and discovered a “do not resuscitate” order, signed by the patient. The paperwork also stated that he had advanced cancer. It was clear why the patient had previously decided he did not want any procedures to be done, which we were quickly prepping to perform. With this added information we put a stop to our efforts, followed the wishes of the patient, and made him as comfortable as possible in his final moments.

My attending asked the paramedics if the patient’s family was coming, and they said they would be arriving in 5–10 minutes. At the time, I was the senior resident in charge, and I was feeling the increased pressure of wanting to see more patients and to get some charting done. A 5–10 minute wait may not seem like much, but in the shift of an emergency physician, it can feel like an eternity. However, my attending didn’t leave the patient’s side, and he held his hand the whole time.

After several long minutes, our patient’s family arrived in the emergency department. We went over the care of the patient and the family quickly understood the entire situation. With tears in their eyes, they still had one request from us: they asked that my attending and I participate with their family in prayer. Of course, we could not refuse. So as we gathered around the bedside, all holding hands, we instantly felt for that brief moment as members of the family. The more we listened to the prayer, something incredible happened. The patient, whom we had no previous connection to and knew nothing about, quickly became very real to us. During the prayer the family described our patient as not only a wonderful, loving person, but also the rock and foundation of this entire family, and that this dying patriarch would be missed beyond measure. What an unforgettable moment that was, to instantly connect to our patient and his family during his last moments on earth.

Then I had an epiphany—what would it have looked like if the family had arrived and no one was at the bedside? It would have looked like their loved one was being abandoned in his final hour. But his family arrived to see my attending holding the hand of their loved one, and he was instantly able to explain the entire situation the moment they arrived. In emergency medicine, we train to save lives. We live for chest tubes, charged paddles, and difficult airways. In reality, the most important and meaningful thing we can do sometimes is as simple as staying in the room a few more minutes.

